# Aortic hemodynamics and white matter hyperintensities in normotensive postmenopausal women

**DOI:** 10.1007/s00415-017-8476-1

**Published:** 2017-04-07

**Authors:** Jill N. Barnes, Ronée E. Harvey, Samantha M. Zuk, Emily S. Lundt, Timothy G. Lesnick, Jeffrey L. Gunter, Matthew L. Senjem, Lynne T. Shuster, Virginia M. Miller, Clifford R. Jack, Michael J. Joyner, Kejal Kantarci

**Affiliations:** 1 Department of Anesthesiology, Mayo Clinic, Rochester, MN USA; 2 Department of Physiology and Biomedical Engineering, Mayo Clinic, Rochester, MN USA; 3 Department of Radiology, Mayo Clinic, 200 1st Street SW, Rochester, MN 55905 USA; 4 Department of Health Sciences Research, Mayo Clinic, Rochester, MN USA; 5 Department of Informational Technology, Mayo Clinic, Rochester, MN USA; 6 Department of Internal Medicine, Women’s Health Clinic, Mayo Clinic, Rochester, MN USA; 7 Department of Surgery, Mayo Clinic, Rochester, MN USA

**Keywords:** MRI, Blood pressure, Cognitive aging, Stroke prevention, Cerebrovascular disease

## Abstract

Hypertension is associated with development of white matter hyperintensities (WMH) in the brain, which are risk factors for mild cognitive impairment. Hormonal shifts at menopause alter vascular function putting women at risk for both hypertension and WMH. Elevations in aortic hemodynamics precede the appearance of clinically defined hypertension but the relationship of aortic hemodynamics to development of WMH in women is not known. Therefore, this study aimed to characterize aortic hemodynamics in relationship to WMH in postmenopausal women. Aortic systolic and diastolic blood pressure (BP), aortic augmentation index (Alx) and aortic round trip travel time (Aortic *T*_R_) by tonometry were examined in 53 postmenopausal women (age 60 ± 2 years). WMH was calculated from fluid-attenuated inversion recovery MRI using a semi-automated segmentation algorithm. WMH as a fraction of total white matter volume positively associated with aortic systolic BP (regression coefficient = 0.018; *p* = 0.04) after adjusting for age. In addition, WMH fraction was positively associated with AIx (0.025; *p* = 0.04), and inversely associated with Aortic *T*_R_ (−0.015; *p* = 0.04) after adjusting for age. Our results suggest that assessing aortic hemodynamics may identify individuals at risk for accelerated development of WMH and guide early treatment to reduce WMH burden and cognitive impairment in the future.

## Introduction

White matter hyperintensities (WMH) in the brain are risk factors for mild cognitive impairment [1, 2] and are associated the rate of cognitive decline in older adults [3]. The volume of WMH is associated with hypertension, especially at midlife [4]. Similarly, arterial stiffness at midlife, which often develops prior to hypertension [5], predicts future WMH volume [6]. Early changes in arterial structure of the central elastic arteries affect aortic hemodynamics prior to clinical changes in brachial BP [7]. Unlike diastolic BP, which is similar when measured centrally (aortic) or peripherally (brachial), systolic BP is dependent on the location in the arterial tree where it is measured [7]. These differences in systolic BP are due to pulse pressure amplification and may have an impact on risk stratification [8]. In fact, some evidence suggests that treatment strategies to mitigate cardiovascular risk should be based on aortic hemodynamics [8, 9]. Thus, it is possible that, even though brachial systolic BP may be within the “normative” range, central or aortic hemodynamics may be associated with vascular brain injury as early as midlife.

Women are at elevated risk for developing hypertension after menopause [10]. However, in postmenopausal women without clinically defined hypertension, the volume of WMH as a fraction of total white matter volume associated with blood-borne markers of endothelial disruption, such as the thrombogenic microvesicles [11] suggesting that changes in WMH volume or fraction precede overt elevations in brachial BP. As there is inadequate examination of potential sex differences in the effects of hypertension and hormonal status on brain structure, this study sought to determine if aortic hemodynamics and arterial stiffness were associated with WMH in a cohort of postmenopausal women.

## Methods

### Participants

Women (*n* = 74) who had previously participated in the Kronos Early Estrogen Prevention Study (KEEPS) at Mayo Clinic were invited to participate in the present study. For participation in KEEPS, women were between 6 months and 3 years past their last menses and without prior cardiovascular events. Menopausal status was confirmed by 17β estradiol and follicular stimulating hormone levels. Full inclusion criteria for KEEPS are reported elsewhere [11, 12]. For the current study, women were recruited 3 years after their exit from the KEEPS study. The current study was to evaluate hemodynamic factors in these postmenopausal women in relationship to the fractional volume of WMH. Inclusion criteria were: (1) body mass index (BMI) <35 kg/m^2^; (2) no history of cardiovascular disease; (3) no diagnosis of diabetes; (4) no prescribed use of antihypertensive medication; (5) systolic brachial BP <150 mmHg and diastolic BP <95 mmHg during both study visits; (6) free of contraindications for MRI for safety such as an MRI-incompatible implant or claustrophobia; and (7) no neurologic diseases present that would have an impact on MRI findings such as multiple sclerosis, brain tumors, or epilepsy. All women underwent a neuropsychological assessment and their scores were within age-adjusted normative ranges [12]. All participants were non-smokers.

### Standard protocol approvals, registrations and patient consents

This study was approved by the Mayo Clinic Institutional Review Board. All participants gave written informed consent.

### Hemodynamic measurements

Participants arrived to the Clinical Research Unit at Mayo Clinic between 11:00 and 13:30 after a 4-h fast and 24 h without caffeine, alcohol, and or exercise. Venous blood was collected for analysis of cardiometabolic risk factors (Table 1). Women rested in the supine position [13] and brachial BP was measured by the cuff method 3 times, each separated by 2 min. Because normotensive individuals sometimes demonstrate higher than normal BP during laboratory study visits, women were excluded if their brachial BP was >150/95 mmHg or if they were using antihypertensive medications. After supine brachial BP measurements, aortic hemodynamics measurements were measured in the supine position. The radial arterial waveforms were calibrated from the supine brachial BP measurements taken immediately prior by an automated oscillometric device (Cardiocap/5, Datex-Ohmeda, Louisville, CO, USA). High-fidelity radial artery pressure waveforms were recorded by applanation tonometry of the radial pulse in the right wrist using a pencil type micromanometer (Millar Instruments, Houston, TX, USA). Multiple trials of sequential radial pulse waveforms were recorded over a 10-s period for each woman. Three to five trials of the radial pulse obtained in rapid succession were averaged for each participant [14]. A generalized transfer function (Sphygmocor, Atcor Medical, Sydney Australia) to correct for upper limb pressure amplification was used to generate the corresponding aortic pressure waveform and central BP. The generalized transfer function has been validated using both intra-arterially [15] and non-invasively obtained radial pressure waves [16].

**Table 1:** Demographic and clinical characteristics

Variables	Median (IQR)
Number of participants	53
Age (years)	60 (59, 61)
Education, no. (%)	
High school diploma	3 (6)
College graduate	32 (64)
Some graduate or professional	2 (4)
Graduate or professional degree	12 (24)
Time past menopause, months	102 (96, 111)
Body mass index (kg/m^2^)	27 (23, 31)
Waist circumference (cm)	87 (79, 96)
Total cholesterol (mg/dL)	204 (190, 226)
Low-density lipoprotein (mg/dL)	120 (104, 136)
High-density lipoprotein (mg/dL)	64 (57, 75)
Triglycerides (mg/dL)	88 (72, 109)
Fasting glucose (mg/dL)	93 (89, 98)
hsCRP (pg/mL)	1.2 (0.5, 2.6)
Estradiol (pg/mL)	5 (3, 10)
Testosterone (ng/dL)	15 (11, 21)
WMH fraction (%)	0.0032 (0.0020, 0.0047)

*hsCRP* C-reactive protein, *WMH* white matter hyperintensities

**Fig. 1:**
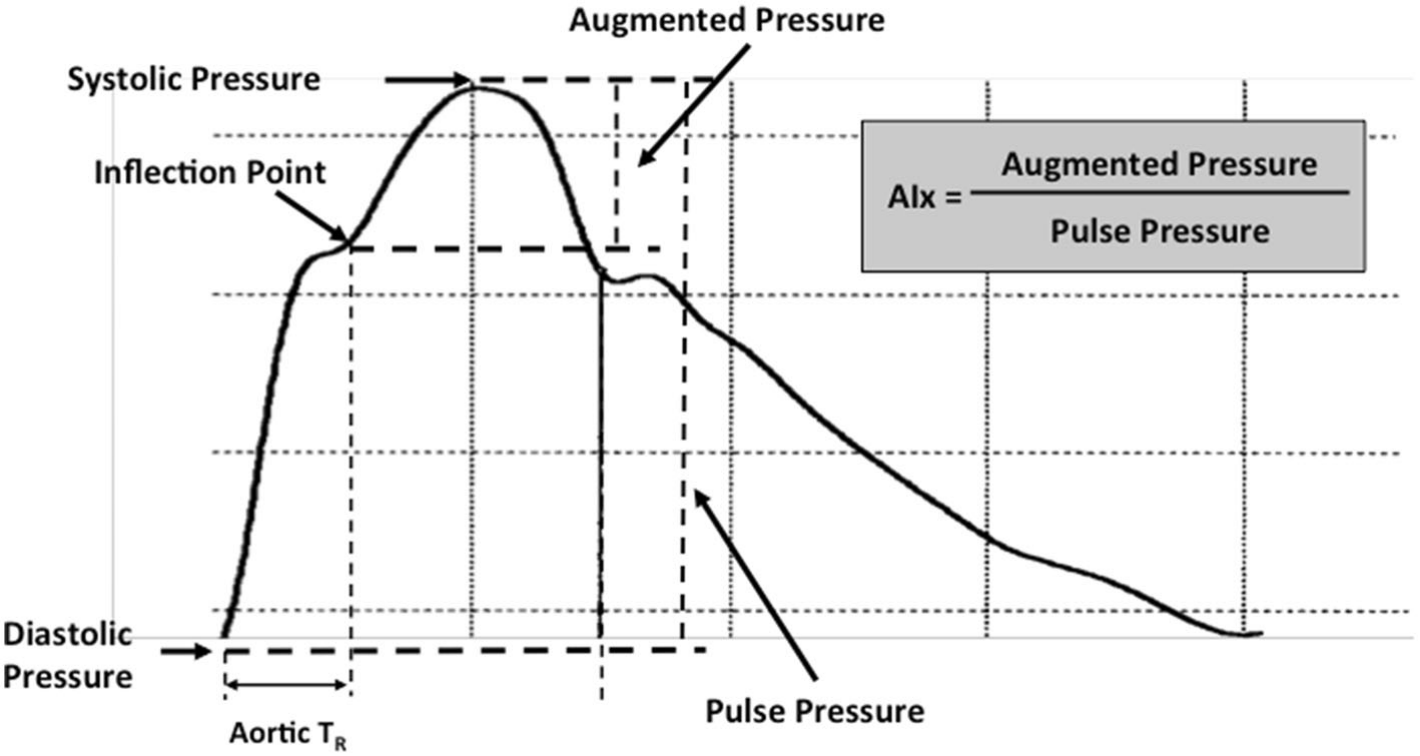
Typical applanation tonometry-derived ascending aortic pressure waveform with pulse wave analysis components including aortic systolic pressure; aortic diastolic pressure; inflection point where incident and reflected waves merge; or round trip travel time of reflected pressure wave to peripheral reflecting sites and back to heart; and AIx or augmentation index, the ratio of augmented pressure to pulse pressure

The central aortic pressure waveform is composed of a forward traveling wave, generated by left ventricular ejection and a reflected wave that is returning to the ascending aorta from the periphery (Fig. 1). Pulse wave analysis of the aortic pressure waveform provided the following variables of interest: (1) central aortic BP; (2) augmented pressure (AP), the amplitude of the reflected wave which is defined as the difference between the first (forward wave) and second systolic shoulder of the aortic systolic BP; (3) AIx, augmentation index, the reflected wave amplitude divided by pulse pressure expressed as a percentage and adjusted for a heart rate of 75 bpm; (3) round trip travel time (Aortic *T*_R_) of the forward traveling wave from the ascending aorta to the major reflection site and back is measured from the beginning of the upstroke of the pressure wave to the foot of the reflected wave (inflection point); and (4) wasted left ventricular energy (LV wasted energy), which is the component of extra myocardial oxygen requirement due to early systolic wave reflection. LV wasted energy can be estimated as 1.333*(π/4)*(augmented pressure * Δ*t*_r_), where 1.333 is the conversion factor and Δ*t*_r_ is the time from the inflection point to the dicrotic notch (systolic duration of the reflected wave). Only high-quality recordings, defined as an in-device quality index of over 80% (derived from an algorithm including average pulse height variation, diastolic variation, and the maximum rate of rise of the peripheral waveform), were accepted for analysis. In general, 3–5 measurements were performed to obtain an acceptable quality index.

### Magnetic resonance imaging

MRI was performed on a single 1.5-tesla system with an 8-channel phased-array head coil (GE Healthcare, Milwaukee, WI, USA) within 6 weeks of the hemodynamic measurements. Fluid-attenuated inversion recovery (FLAIR) MRI of the whole head was performed to quantify WMH volume. A 3-dimensional magnetization-prepared rapid acquisition gradient echo (MPRAGE) sequence was used for the segmentation of WM. All MRIs underwent preprocessing corrections for gradient nonlinearity and intensity nonuniformity.

WMH volumes were derived from a semi-automated segmentation of FLAIR images as detailed in previously published work [11]. Briefly, All MPRAGE and FLAIR images obtained during the same examination period were co-registered and segmented, and MPRAGE image was resampled in the FLAIR space. WMH on FLAIR images were segmented using an automated slice-based seed initialization and region-growing method, and the segmented WMH voxels were multiplied with the WMH mask. A trained image-analyst (S.Z.) inspected the segmented WMH mask overlaid on the FLAIR image. Every segmented slice was visually compared with the unprocessed FLAIR images and all false-positive WMH labels that resulted from artifacts were edited and excluded from the WMH mask [11]. To adjust for potential differences in WM volume among women, WMH fraction was calculated as WMH volume/total WM volume to determine the fraction of WM at risk.

### Data analysis and statistics

A paired *t* test was performed to determine if aortic and brachial systolic BP values were statistically different. In all analyses, WMH fraction was log-transformed to improve normality and reduce skewness. To assess the relationship between WMH fraction and aortic hemodynamic measures, we used linear regression models predicting log (WMH fraction) with each aortic hemodynamic measure taken one at a time while adjusting for age. The *t* test and regression analyses were performed using statistical analysis software R version 3.1.1.

## Results

### Participant characteristics

Of the 74 women who agreed to participate in this study, twenty-one women were excluded from analysis due to a BMI >35 kg/m^2^ (*n* = 8), presence of uncontrolled hypertension (*n* = 4), use of anti-hypertensive medication (*n* = 8), and feeling claustrophobic in the MRI scanner (*n* = 1). The demographic and clinical characteristics of the 53 women meeting inclusion criteria are shown in Table 1.

### Hemodynamic variables

Median brachial and aortic systolic and diastolic pressures, IQR values for augmented pressure, AIx (adjusted for heart rate), Aortic *T*_R_ and LV wasted energy are listed in Table 2.

**Table 2:** Brachial and aortic hemodynamics

Variables	Median (IQR)
Brachial SBP (mmHg)	123 (115, 127)
Brachial DBP (mmHg)	73 (69, 79)
Brachial PP (mmHg)	38 (34, 42)
Aortic SBP (mmHg)	112 (104, 119)
Aortic DBP (mmHg)	74 (69, 78)
Augmented pressure (mmHg)	13 (10, 16)
AIx (%)	27 (23, 31)
Aortic *T*_R_ (ms)	141 (133, 146)
LV wasted energy (dynes cm^2^ s)	2.8 (2.2, 3.4)

LV wasted energy is in thousands

*AIx* aortic augmentation index at a heart rate of 75 bpm, *Aortic T*_*R*_ round trip travel time, an indirect measure of aortic pulse wave velocity, *DBP* diastolic blood pressure, *LV* left ventricle, *PP* pulse pressure, *SBP* systolic blood pressure

### Associations between WMH and aortic hemodynamic characteristics

**Fig. 2:**
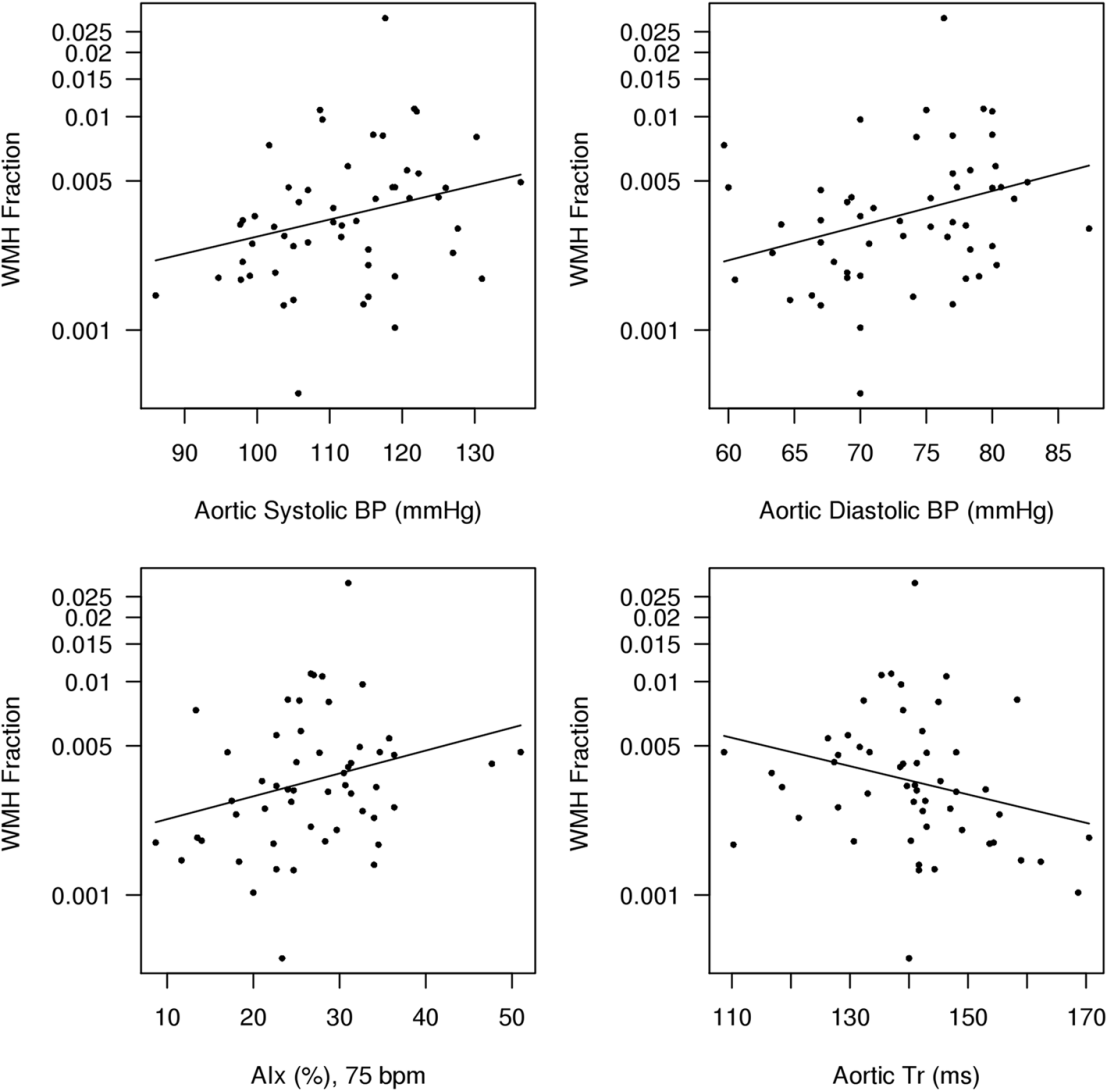
Scatterplot and regression line for aortic hemodynamic variables and white matter hyperintensity (WMH) fraction. The regression models include an additive age-effect and the regression line is shown for the mean 60 years of age. *AIx* aortic augmentation index at a heart rate of 75 bpm, *Aortic T*_*R*_ round trip travel time, an indirect measure of aortic pulse wave velocity, *DBP* diastolic blood pressure, *SBP* systolic blood pressure

WMH fraction was positively associated with aortic systolic BP and AIx, and inversely associated with Aortic *T*_R_ (Table 3; Fig. 2). There was no association between WMH fraction and aortic diastolic BP, augmented pressure, or LV wasted energy (*p* > 0.05 for all; Table 3). Eliminating the effect of potential outliers (highest and lowest WMH fraction) did not change the results or statistical significance (data not shown).

**Table 3:** Regression model estimates (SE) between log (WMH fraction) and hemodynamic variables adjusted for age

Variable	Regression coefficient (SE)	% change in WMH fraction for 1-unit increase in variable	*p* value
Brachial systolic BP (mmHg)	0.018 (0.01)	1.8	0.08
Brachial diastolic BP (mmHg)	0.024 (0.015)	2.4	0.11
Pulse pressure (mmHg)	0.013 (0.012)	1.3	0.27
Aortic systolic BP (mmHg)	0.018 (0.009)	1.8	0.046
Aortic diastolic BP (mmHg)	0.028 (0.015)	2.8	0.07
Augmented pressure (mmHg)	0.032 (0.019)	3.2	0.10
AIx (%) @ 75 bpm	0.025 (0.011)	2.5	0.04
Aortic *T*_R_ (ms)	−0.015 (0.0073)	−1.5	0.04
LV EW in thousands	0.1 (0.072)	10	0.17

Regression model beta estimates (standard error) between log (WMH fraction) and hemodynamic variables. All estimates were age-adjusted. Intercept estimates are not shown

*AIx* aortic augmentation index at a heart rate of 75 bpm, *Aortic T*_*R*_ round trip travel time, an indirect measure of aortic pulse wave velocity, *BP* blood pressure, *LV EW* left ventricular wasted energy

### Associations between WMH and brachial BP

The median brachial systolic BP was significantly greater than the median aortic systolic BP (*p* < 0.0001; Table 2); but median aortic diastolic BP did not differ from brachial diastolic BP (*p* > 0.05). There was a non-significant trend for an association between WMH fraction and brachial systolic BP (*p* = 0.08, Table 3). The age-adjusted association between WMH and brachial diastolic BP and brachial PP did not reach significance (*p* = 0.11, *p* = 0.27, respectively; Table 3).

### Association of WMH with other confounders

WMH fraction did not associate with BMI or with MAP. Adjusting for height did not change the association between WMH fraction and aortic variables (data not shown).

## Discussion

This study demonstrated that WMH fraction associated with aortic hemodynamic characteristics in postmenopausal women who were not using antihypertensive medications and who did not meet the clinical criteria for hypertension. Non-invasive pulse wave analysis has been used to detect changes in aortic characteristics for decades. The pressure waveform at the central aorta is important for assessing cardiovascular risk because it represents the work imposed on the left ventricle and central elastic arteries [17]. A physiological increase in AIx reflects arterial stiffening, a change in downstream reflection sites, or elevated peripheral resistance. Similarly, a decrease in the time of reflection (Aortic *T*_R_) characterizes an increase in arterial stiffness. Unfavorable increases in aortic BP and AIx are associated with cardiovascular diseases such as hypertension, stroke, and coronary heart disease [18]. The present results expand these observations to show that the aortic parameters are also related to WMH burden in women who do not meet the current clinical definition for hypertension based on brachial arterial blood pressure. Therefore, assessing aortic hemodynamics and arterial stiffness in postmenopausal women with systemic brachial BP within what is considered pre-hypertensive ranges may be important in identifying risk for WMH, and thus enable targeted prevention strategies.

A higher volume of WMH is associated with cardiovascular disease and risk factors such as age, myocardial infarction, hypertension, and atherosclerosis [19, 20]. During the transition to and after menopause, the risk of developing hypertension, ischemic heart disease, and stroke increases in women [21]. The decreases in ovarian hormones, in particular the loss of estrogen, during the transition to menopause may accelerate large artery stiffness, which can be detected in individuals with normal brachial BP values [5, 7, 22]. Indeed, sex differences in blood pressure parameters observed between young women and men [23], are not observed when postmenopausal women are compared to age-matched men [23, 24]. Therefore, aortic arterial hemodynamics may be a more sensitive measure of evaluating elevated risk for both cardiovascular and cerebrovascular disease than brachial BP.

The central elastic arteries are capable of transmitting pulsatile flow and cushion this pulsatility to smooth laminar flow in the peripheral arteries and microvessels. However, due to the short distance between the central elastic arteries and cerebral vasculature, the brain may be exposed to high pulsatile flow if this cushioning function is lost. A slight increase in arterial stiffness disrupts the ability of the elastic arteries to dampen the high pulsatility, so this can be transmitted to the cerebral vasculature. Whereas peripheral arteries are often protected from damage due to pulsatility because of downstream vasoconstriction, the brain is a high flow system with overall low impedance. The combination of the short distance between the central elastic arteries and the brain, as well as the high blood flow needs of the tissue, makes the brain particularly susceptible to damage from arterial stiffness [25]. Therefore, a small change in aortic hemodynamics or arterial stiffness can have a substantial impact on the microvasculature in the brain [25–27]. Indeed, in the present study, AIx and Aortic *T*_R_ measurements correlated with WMH fraction an observation that is consistent with the idea that pulse wave analysis provides a comprehensive assessment of the vascular stress placed on the cerebral circulation. The North American Artery Society recommends that pulse wave analysis be incorporated in guiding BP treatments in pre-hypertensive adults [27]. In addition, a recent study concluded that arterial stiffness predicted cognitive decline in healthy adults better than brachial BP [28]. Our results suggest that managing arterial stiffness and aortic BP, may be useful to reduce cardiovascular risk, and to reducing WMH burden that is associated with cognitive decline.

Previous studies in men and women have shown a positive correlation between aortic pulse wave velocity, a measure of arterial stiffness, and WMH volume. For example, pulse wave velocity associated with WMH in hypertensive adults [29], patients with recent minor stroke or transient ischemic attack [30], and patients without stroke or dementia in the Framingham Offspring Study [31]. A recent study demonstrated that even in young adults (age 30–45 years), aortic pulse wave velocity was associated with WMH volume [32]. Furthermore, aortic pulse wave velocity is associated with the WMH load measured after 10 years, suggesting that early increases in pulse wave velocity may precede WMH development 10 years later [6]. Additional follow-up of the women in our study is needed to better establish the clinical relevance between these hemodynamic measures, brain structure and cognition.

The main limitation of the present study is that the age range of the participants was narrow and the women had low risk cardiometabolic profiles. In addition, because this cohort consisted of white postmenopausal women, these findings cannot be generalized to postmenopausal women of different ethnic backgrounds, premenopausal women or men. Another limitation is that with this small sample, there was insufficient statistical power to adjust for all potential confounding variables. Despite these limitations, these findings suggest that even in postmenopausal women without clinically defined hypertension, higher aortic systolic BP, greater wave augmentation, and faster Aortic *T*_R_ are associated with greater WMH fraction. Because increases in aortic hemodynamics and arterial stiffness often precede changes in brachial BP measurements [5, 7], these results suggest that assessing aortic hemodynamics may identify normotensive women at increased risk for WMH and guide interventions to reduce development of WMH. Longitudinal evaluation of the participants is critical for determining the influence of WMH on cognitive outcomes.
